# LPS and β-Glucan Induce Differential Memory-like Inflammatory Responses with Enhanced Reactivity in Neonatal Neutrophils Compared to Adults

**DOI:** 10.3390/ijms27167268

**Published:** 2026-08-14

**Authors:** Lisa Zagler, Laura Snaidr, Natascha Köstlin-Gille, Heidi Hildenbrand, Nadja Kliegel, David L. Williams, Ricarda Will, Stefanie Dietz-Ziegler, Cahit Birdir, Christian Gille, Trim Lajqi

**Affiliations:** 1 Department of Neonatology, Medical Faculty Heidelberg, University of Heidelberg, 69120 Heidelberg, Germany; 2 Department of Anaesthesiology, Medical Faculty Heidelberg, University of Heidelberg, 69120 Heidelberg, Germany; 3 Department of Surgery and Center of Excellence in Inflammation, Infectious Disease and Immunity, Quillen College of Medicine, East Tennessee State University, Johnson City, TN 37614, USA; 4 Department of Gynaecology and Obstetrics, Medical Faculty Heidelberg, University of Heidelberg, 69120 Heidelberg, Germany

**Keywords:** neutrophils, memory-like, adult, neonates, inflammation, metabolism, signaling

## Abstract

Neutrophils are key effectors of innate immunity. Although traditionally regarded as short-lived effector cells, recent evidence suggests they can undergo functional reprogramming after microbial stimulation, leading to trained immunity. We investigated whether lipopolysaccharide (LPS) or *Candida albicans* β-glucan induces memory-like inflammatory responses in adult peripheral and neonatal cord blood neutrophils. We used complementary molecular and functional approaches to characterize neutrophil responses following *in vitro* priming with LPS or β-glucan and subsequent LPS restimulation. Cytokine secretion, reactive oxygen species (ROS) production, glycolytic activity (via lactate production and glycolytic enzyme expression), and ERK1/2 and NF-κB signaling pathway activation were assessed. Priming with LPS or β-glucan enhanced neutrophil responsiveness to secondary stimulation, resulting in increased secretion of IL-1β, IL-6, IL-8, CXCL1, and CCL2, together with elevated ROS production. These effects were consistently more pronounced in neonatal than in adult neutrophils. Memory-like neutrophils also exhibited metabolic reprogramming, associated by increased hexokinase-2 and phosphofructokinase-1 mRNA expression, elevated lactate production, and enhanced ERK1/2 and NF-κB activation. Human neutrophils, particularly neonatal cells, develop memory-like characteristics following LPS or β-glucan priming, highlighting their functional and metabolic plasticity and suggesting a role for neutrophil training in early-life immune adaptation.

## 1. Introduction

Neutrophils are short-lived, terminally differentiated immune cells and represent the most abundant leukocyte population in human blood. They play a major role in innate immune defense and are among the first cells recruited to sites of infection or tissue injury [1,2,3]. In response to microbial invasion, neutrophils rapidly activate a broad range of antimicrobial mechanisms, including the secretion of cytokines and chemokines, generation of ROS, degranulation, phagocytosis, and formation of neutrophil extracellular traps (NETs) [4,5]. In recent years, their function has been recognized as extending beyond immediate pathogen clearance, since neutrophils can also influence chronic inflammatory processes and regulate interactions with other immune cells [2,6]. Although neutrophils are generally considered short-lived cells, their survival can increase considerably under inflammatory conditions, allowing them to remain active within affected tissues for prolonged periods [7,8,9].

The neonatal immune system differs substantially from that of adults and still retains several characteristics associated with fetal immunity. Early-life immune responses are often skewed toward anti-inflammatory regulation, which contributes to the increased susceptibility of newborns to infection. This reduced immune responsiveness has been linked, among other factors, to lower production of interferon-γ (IFN-γ) and IL-12 by T lymphocytes [10,11,12,13]. Development of neutrophils begins during the first trimester of pregnancy, approximately around gestational week 8, followed by rapid granulopoiesis after birth that results in comparatively high circulating neutrophil numbers in neonates [13,14]. Although neonatal neutrophils show weaker chemotactic responses and lower phagocytic efficiency, the marked postnatal increase in neutrophil numbers is likely an important protective mechanism that helps newborns cope with the sudden exposure to microorganisms after delivery.

Although immunological memory was traditionally considered exclusive to adaptive immunity, accumulating evidence demonstrates that innate immune cells can also develop long-lasting functional (memory-like) reprogramming following an initial stimulus [15,16,17]. This phenomenon, referred to as trained immunity, involves epigenetic as well as metabolic alterations, including a shift toward enhanced glycolysis [14,16]. Trained immunity may develop centrally in the bone marrow during granulopoiesis or peripherally in circulating cells [15,18,19]. Various pathogen-associated molecular patterns (PAMPs), including β-glucan from *Candida albicans* and Bacille Calmette-Guérin (BCG), as well as endogenous danger signals such as oxidized low-density lipoprotein (oxLDL) and heme, have been shown to induce trained immunity characterized by enhanced production of pro-inflammatory mediators, including IL-1β, IL-6, and ROS [15,16,20,21]. Despite their short lifespan, neutrophils have also been reported to exhibit memory-like responses. Several studies demonstrated that inflammatory mediators such as TNF-α and IL-8 in human neutrophils, as well as LPS and lipoteichoic acid (LTA) in murine neutrophils, promote enhanced pro-inflammatory activity together with improved recruitment and antimicrobial functions [22,23,24,25]. In newborns, whose immune defense relies predominantly on innate immunity, the development of memory-like responses may be particularly important for protection against invading pathogens.

In the present study, we investigated whether bacterial LPS and β-glucan (*Candida albicans*) induce memory-like inflammatory responses in adult and neonatal human neutrophils and characterized the associated inflammatory, metabolic, and signaling alterations *in vitro*. We further compared the responses of neonatal and adult neutrophils to determine whether developmental age influences the magnitude of these responses.

## 2. Results

### 2.1. Short-Term LPS and β-Glucan Priming Induces Pro-Inflammatory Cytokine and Chemokine Release in Adult and Neonatal Neutrophils

**Figure 1:**
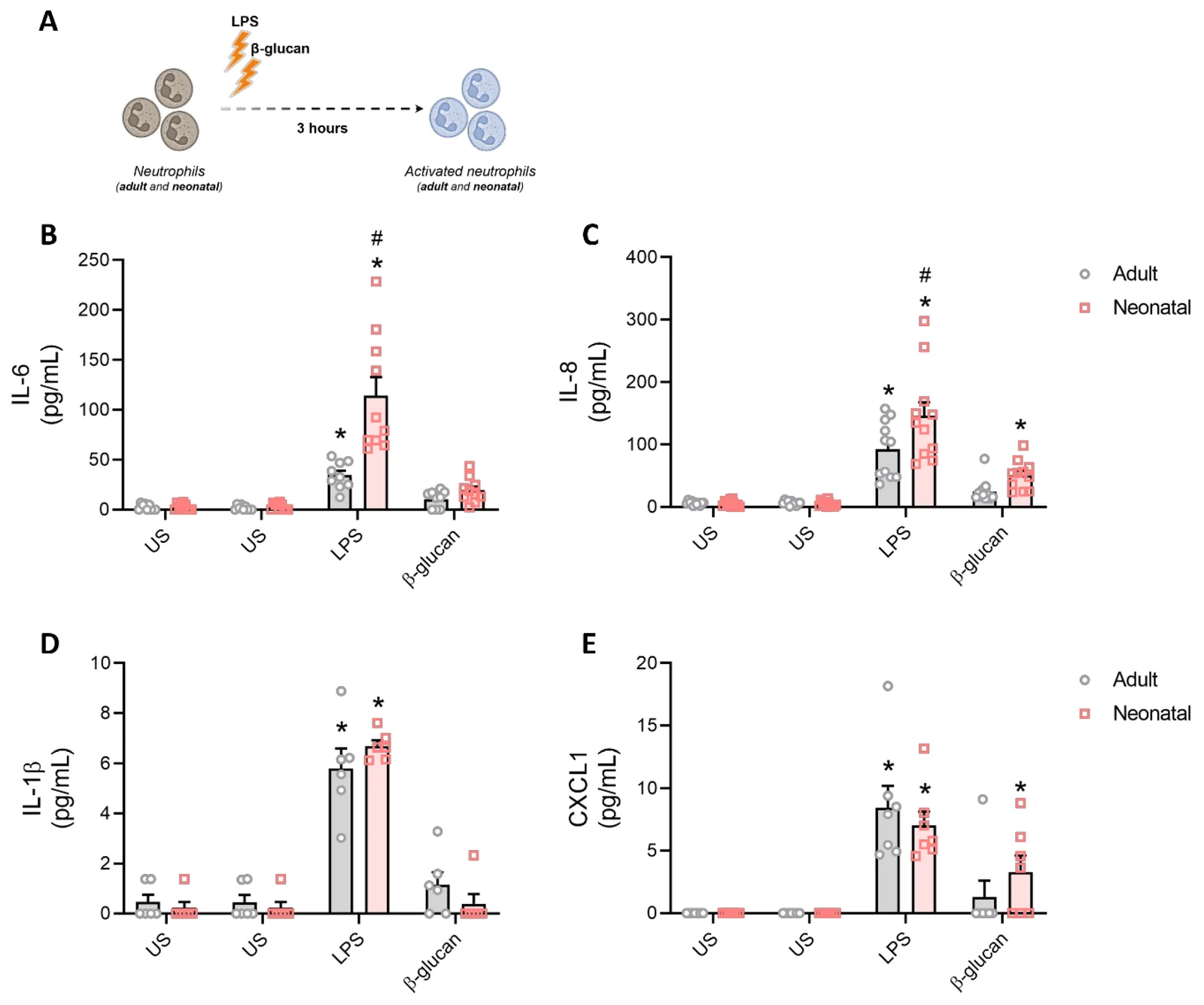
Early inflammatory responses of adult and neonatal neutrophils following primary stimulation with LPS or β-glucan. (**A**) Experimental scheme illustrating the 3 h priming protocol used for stimulation of isolated human adult and neonatal neutrophils with LPS (100 ng/mL) or β-glucan (10 µg/mL). Cytokine and chemokine levels in culture supernatants was subsequently quantified using ELISA. Concentrations of (**B**) IL-6 (n = 9–10), (**C**) IL-8 (n = 11), (**D**) IL-1β (n = 6), and (**E**) CXCL1 (n = 7) produced by adult and neonatal neutrophils after stimulation are shown. Data were presented as scatter dot plots, mean ± SEM. Data were analyzed using a mixed-effects model (REML) with Šidák-adjusted multiple comparisons. * *p* < 0.05, * indicating statistical significance compared to unstimulated (US) control and # *p* < 0.05, # indicating statistical significance compared to adult group.

To characterize the immediate inflammatory responsiveness of adult and neonatal neutrophils, cells were subjected to a short-term priming phase of 3 h with either LPS or β-glucan, as outlined in the experimental scheme (Figure 1A). Following stimulation, the release of key pro-inflammatory mediators, including IL-6, IL-8, IL-1β, and CXCL1, was quantified in culture supernatants (Figure 1B–E).

Both LPS and β-glucan (*Candida albicans*) robustly induced cytokine and chemokine production in neutrophils from adult peripheral blood as well as from neonatal cord/placental blood, indicating that both cell populations are capable of rapid inflammatory activation upon primary stimulation. Overall, exposure to either stimulus resulted in a marked increase in all measured inflammatory mediators compared with baseline US conditions. Notably, neonatal neutrophils exhibited a different response pattern following LPS stimulation. Specifically, the production of IL-6 and IL-8 was significantly elevated in neonatal cells compared with adult neutrophils (*p* < 0.001), indicating that neonatal neutrophils may possess an increased ability to produce certain pro-inflammatory mediators during the early phase of activation (Figure 1B,C). In contrast, this pattern was not consistent for all measured analytes, as stimulation-dependent induction of IL-1β and CXCL1 was similar between neonatal and adult neutrophils.

### 2.2. LPS or β-Glucan Priming Induces a Memory–like Phenotype in Adult and Neonatal Neutrophils Following Secondary LPS Challenge

**Figure 2:**
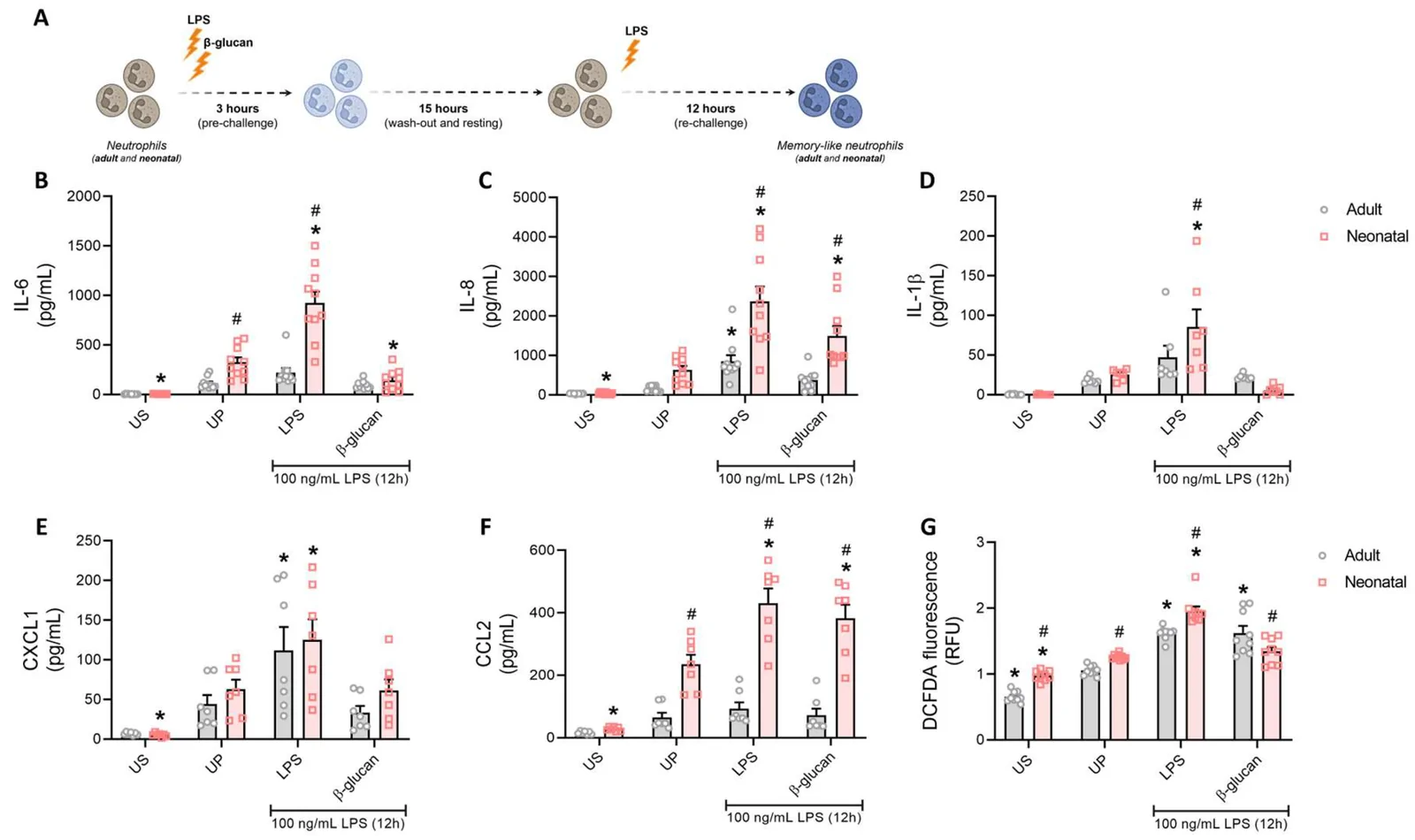
Memory-like inflammatory responses in adult and neonatal neutrophils following secondary stimulation. (**A**) Schematic representation of the experimental protocol used to induce memory-like responses in human neutrophils. Cells were initially primed with LPS (100 ng/mL) or β-glucan (10 µg/mL) for 3 h, followed by a resting period of approximately 15 h and subsequent restimulation with LPS (100 ng/mL) for 12 h. Production of (**B**) IL-6 (n = 10), (**C**) IL-8 (n = 10), (**D**) IL-1β (n = 7), (**E**) CXCL1 (n = 7), and (**F**) CCL2 (n = 7) was quantified in culture supernatants by ELISA. (**G**) Intracellular ROS (n = 3 independent experiments, each with 3 technical replicates) generation was assessed using the DCFDA/H2DCFDA—Cellular ROS assay, and presented as relative fluorescence units (RFU). Data were presented as scatter dot plots, mean ± SEM. Data were analyzed using a mixed-effects model (REML) with Šidák-adjusted multiple comparisons. * *p* < 0.05, * indicating statistical significance compared to unprimed (UP) control and # *p* < 0.05, # indicating statistical significance compared to adult group.

To investigate whether primary microbial stimulation promotes memory-like inflammatory responses in human neutrophils, adult and neonatal cells were primed with LPS or β-glucan for 3 h, followed by a resting phase of approximately 15 h and subsequent restimulation with LPS (100 ng/mL) for 12 h, as illustrated in Figure 2A. Prior to performing the main experiments, an LPS dose titration (1, 10, and 100 ng/mL) was conducted in adult control neutrophils to evaluate the effect of priming concentration on the initial response and subsequent responsiveness following secondary LPS challenge (Supplementary Figure S2). Based on these results, 100 ng/mL LPS was selected as the priming concentration for subsequent experiments. Production of inflammatory cytokines, chemokines, and ROS was subsequently assessed (Figure 2B–G).

LPS-primed neutrophils displayed enhanced secondary responses compared with UP controls, characterized by increased secretion of IL-6, IL-8, IL-1β, CXCL1, and CCL2, together with elevated ROS generation (*p* < 0.05), consistent with a memory–like phenotype. β-glucan priming also altered neutrophil responsiveness upon secondary LPS challenge, although the effects differed between adult and neonatal cells. In neonatal neutrophils, β-glucan significantly enhanced IL-6, IL-8, and CCL2 production following restimulation (*p* < 0.05), whereas adult neutrophils mainly exhibited increased ROS generation.

Comparison between adult and neonatal neutrophils revealed a more pronounced inflammatory phenotype in neonatal cells after secondary stimulation. With the exception of CXCL1, neonatal neutrophils produced significantly higher levels of IL-6, IL-8, IL-1β, CCL2, and ROS than adult neutrophils under the respective primed conditions (Figure 2B–G; *p* < 0.05). Moreover, IL-8 and IL-1β production in primed but unchallenged adult control neutrophils returned to near-baseline levels following the resting period (Supplementary Figure S3). To determine whether these differences were associated with alterations in cell integrity or protein content, total protein expression, cell viability, and cytotoxicity were additionally evaluated across all experimental groups (Supplementary Figure S4). Aligned rank transform (ART) analysis revealed no significant Group × Condition interaction for Trypan Blue viability (*p* = 0.382), cytotoxicity (*p* = 0.306), or resazurin-based viability (*p* = 0.910), indicating that the effects of the experimental conditions did not differ significantly between adult and neonatal cells. Together with the absence of a significant interaction in total protein content, these findings support the interpretation that the observed inflammatory and oxidative responses were unlikely to be explained by differential effects of the experimental conditions on cell viability, cytotoxicity, or total protein content between the two groups.

### 2.3. Glycolytic Reprogramming Supports Memory-like Responses in Adult and Neonatal Neutrophils

**Figure 3:**
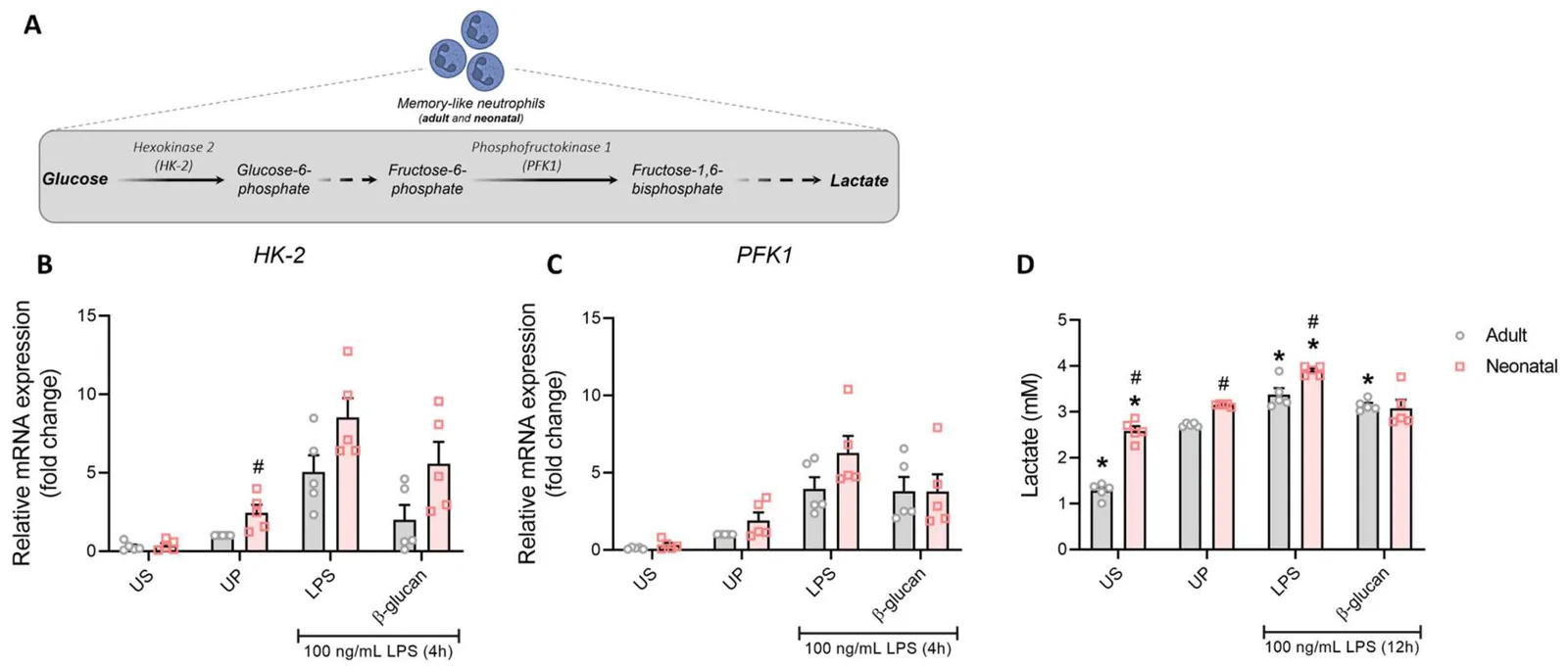
Metabolic rewiring of glycolysis in primed adult and neonatal neutrophils upon restimulation. (**A**) Schematic overview of the glycolytic pathway from glucose uptake to lactate production, highlighting the key enzymes analyzed in this study, hexokinase 2 (HK-2) and phosphofructokinase 1 (PFK1). Cells were initially primed with LPS (100 ng/mL) or β-glucan (10 µg/mL) for 3 h, followed by a resting period of approximately 15 h and subsequent restimulation with LPS (100 ng/mL) for 6 h for RNA isolation for real-time PCR analysis and 12 h for collection of supernatants for lactate measurement. (**B**,**C**) Relative gene expression of HK-2 (n = 5) and PFK1 (n = 5) in adult and neonatal neutrophils assessed by quantitative real-time PCR, with expression normalized to the unprimed adult group (set to 1.0). (**D**) Lactate production (n = 5) measured in cell culture supernatants using a commercially available L-Lactate Assay Kit. Data are presented as scatter dot plots, mean ± SEM. (**B**,**C**) were analyzed using ART, followed by Mann–Whitney U and Wilcoxon signed-rank tests for pairwise comparisons. (**D**) was analyzed using a mixed-effects model (REML) with Šidák-adjusted multiple comparisons. * *p* < 0.05, * indicating statistical significance compared to unprimed (UP) control and # *p* < 0.05, # indicating statistical significance compared to adult group.

Because memory-like responses in innate immune cells are closely linked to immunometabolic adaptation, we next examined whether primed neutrophils undergo glycolytic reprogramming following secondary stimulation. A schematic overview of the glycolytic pathway and the analyzed metabolic targets is shown in Figure 3A. Expression of the key glycolytic enzymes hexokinase 2 (HK-2) and phosphofructokinase 1 (PFK1) was assessed by quantitative real-time PCR, together with measurement of lactate production as a functional indicator of glycolytic activity (Figure 3B–D). Both adult and neonatal neutrophils exposed to LPS or β-glucan priming showed a trend toward increased expression of HK-2 and PFK1 following secondary LPS stimulation compared with UP controls, although these differences did not reach statistical significance (Figure 3B,C). The increase in inflammatory activity was paralleled by higher lactate secretion, supporting the idea that primed neutrophils undergo metabolic adaptation toward glycolysis (Figure 3D; *p* < 0.05).

To further distinguish baseline developmental differences from priming-induced metabolic and inflammatory alterations, unstimulated adult and neonatal neutrophils were compared across all measured parameters (Supplementary Table S1). Neonatal neutrophils showed significantly higher basal lactate levels, CCL2 production, and ROS generation, whereas IL-6, IL-8, IL-1β, and CXCL1 levels were comparable between groups.

A clear difference between neonatal and adult neutrophils became apparent under primed conditions. After LPS priming, neonatal cells expressed higher levels of HK-2 and PFK1 and released more lactate than adult neutrophils treated in the same manner (Figure 3B–D). In addition, neonatal neutrophils already showed elevated lactate concentrations before stimulation, suggesting intrinsic metabolic differences between neonatal and adult cells that are not solely caused by the priming process.

### 2.4. Enhanced ERK1/2, AKT, and NF-κB Activation Accompanies Memory-like Immune Response in Adult and Neonatal Neutrophils

**Figure 4:**
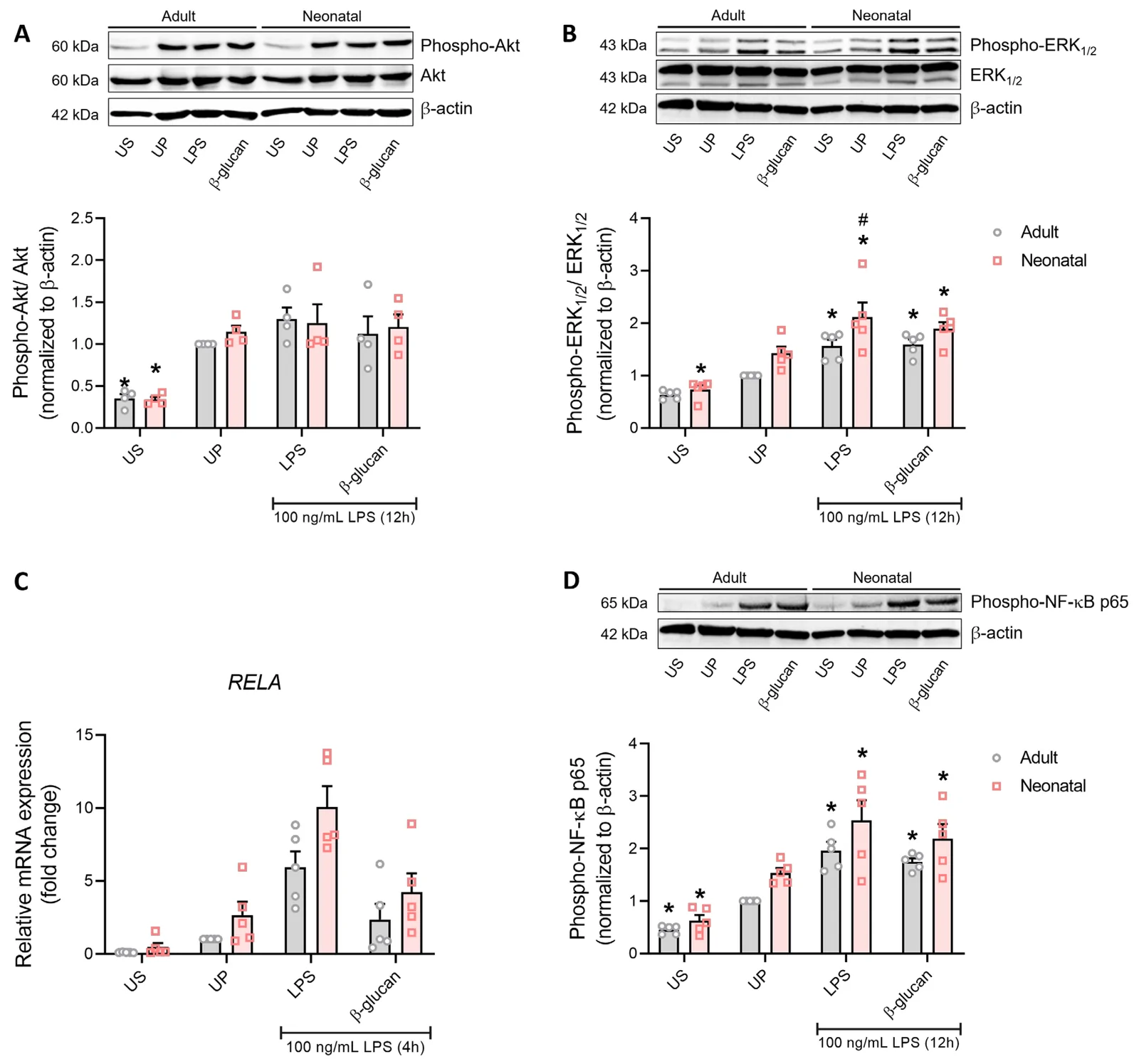
Intracellular signaling pathways underlying memory-like activation in adult and neonatal neutrophils following secondary LPS stimulation. Cells were initially primed with LPS (100 ng/mL) or β-glucan (10 µg/mL) for 3 h, followed by a resting period of approximately 15 h and subsequent restimulation with LPS (100 ng/mL) for 12 h. Cell lysates were analyzed by Western blot for (**A**) phospho-Akt (n = 4), (**B**) phospho-ERK1/2 (n = 5), and (**D**) phospho-NF-κB p65 (n = 5), with expression normalized to the unprimed adult condition (set to 1.0). (**C**) RELA (n = 5) mRNA expression was assessed by quantitative real-time PCR in samples collected 6 h after restimulation with LPS, with normalization to the unprimed adult group (set to 1.0). Data are presented as scatter dot plots, mean ± SEM. (**C**) was analyzed using ART, followed by Mann–Whitney U and Wilcoxon signed-rank tests. (**A**,**B**,**D**) were analyzed using mixed-effects models (REML) with Šidák-adjusted multiple comparisons. * *p* < 0.05, * indicating statistical significance compared to unprimed (UP) control and # *p* < 0.05, # indicating statistical significance compared to adult group. (**B**) (phospho-ERK1/2/ERK1/2) and D (phospho-NF-κB p65) were detected on the same membrane and therefore share the same β-actin loading control. Representative immunoblots from independent biological replicates are shown; the phospho-Akt blot shown in (**A**) represents one biological replicate and is not intended to reflect the magnitude of the overall quantitative response. Quantitative analyses were performed using densitometric measurements from all independent biological replicates indicated above.

To better understand the signaling pathways associated with memory-like responses, intracellular inflammatory signaling was examined in neutrophils from both adults and neonates. Western blot experiments showed increased phosphorylation of protein kinase B (AKT) in cells exposed to LPS or β-glucan compared with unprimed controls in both groups (Figure 4A). Although the same pattern was observed repeatedly, the differences did not reach statistical significance.

More distinct changes were detected within the mitogen-activated protein kinase (MAPK) pathway. Expression of phosphorylated ERK1/2 increased markedly after priming with LPS or β-glucan in adult as well as neonatal neutrophils when compared with unprimed cells (Figure 4B; *p* < 0.05), paralleling the enhanced memory-like inflammatory responses observed following secondary stimulation.

Because NF-κB is a major regulator of inflammatory gene expression, RELA mRNA and phosphorylated NF-κB p65 protein expression were analyzed. Primed neutrophils showed a trend toward higher RELA mRNA expression together with significantly increased phosphorylated NF-κB p65 protein after stimulation with LPS or β-glucan relative to unprimed controls (Figure 4C,D; *p* < 0.05).

The signaling responses were generally stronger in neonatal neutrophils, especially regarding phospho-ERK1/2 and phospho-NF-κB p65 expression. This pattern was consistent with the higher cytokine and chemokine release observed in neonatal cells and was associated with an increased inflammatory responsiveness following microbial priming.

## 3. Discussion

Neonatal immunity has long been described as functionally immature and predominantly anti-inflammatory, which is commonly considered one reason why newborns are particularly vulnerable to severe infections. This assumption mainly originates from studies reporting weaker adaptive immune responses during early life, including reduced T-helper 1 (Th1) polarization and less efficient antigen presentation [10,26,27]. However, accumulating evidence suggests that innate immune responses in neonates are not universally suppressed but are instead selectively regulated depending on the nature and intensity of stimulation [28,29]. Compared with adult neutrophils, neonatal neutrophils frequently show reduced migratory capacity, differences in neutrophil extracellular trap (NET) formation, and lower oxidative burst activity. Nevertheless, they remain highly sensitive to inflammatory signals and represent one of the dominant immune cell populations directly after birth [30,31,32]. In addition to their established antimicrobial role, neutrophils are now recognized as important regulators of immune responses through cytokine secretion and interaction with other leukocyte populations [33,34,35]. Taken together, these observations suggest that neonatal innate immunity is developmentally specialized rather than merely impaired and support the idea that inflammatory stimulation early in life may result in sustained functional adaptation resembling innate immune memory [36,37,38].

The inherently short lifespan of circulating neutrophils, which usually do not survive beyond 48 h in vitro, presents a challenge for studying prolonged functional adaptations in these cells. To overcome this limitation, we used a sequential stimulation approach involving transient priming, a resting interval, and secondary stimulation, in line with previously published models [24,39,40]. Brief exposure to LPS or β-glucan was sufficient to trigger inflammatory activation in neutrophils, as demonstrated by enhanced release of IL-6, IL-8, IL-1β, and CXCL1. Consistent with this observation, Guthrie et al. previously demonstrated that less than one hour of LPS exposure can trigger inflammatory responses in neutrophils [25,41,42]. LPS and β-glucan are recognized by distinct pattern-recognition receptors (PRRs), primarily TLR4 and Dectin-1, and are established initiators of innate immune activation during bacterial and fungal infection [43,44,45]. In addition to their acute effects, both stimuli have been implicated in the induction of memory-like reactions, a process in which prior microbial exposure modifies subsequent innate immune responses through persistent cellular reprogramming [15,16]. In line with this concept, our results show that transient priming with either stimulus augments secondary neutrophil responses, indicating the capacity of these cells for memory-like functional adaptation.

Memory-like neonatal neutrophils showed broadly enhanced cytokine (e.g., IL-6, IL-1β), chemokine (e.g., IL-8, CXCL1, CCL2), and ROS production, particularly following LPS priming, while β-glucan effects were more selective. Adult neutrophils displayed more limited training, mainly affecting IL-8, CXCL1, and ROS. Overall, neonatal neutrophils exhibited stronger responses than adults, except for CXCL1. Furthermore, the return of cytokine production to near-baseline levels after the resting period indicates that the enhanced responses following secondary stimulation were unlikely to result from persistent activation after priming. Persistent ROS elevation may also influence regulated cell death pathways, including apoptosis and pyroptosis [46]. Although no differences in neutrophil viability or cytotoxicity were detected in our model, the contribution of such pathways to memory-like inflammatory responses remains to be clarified. Although endotoxin exposure has historically been associated with tolerance and reduced responsiveness under certain experimental conditions, increasing evidence suggests that the outcome of LPS stimulation depends on multiple factors including concentration, duration of exposure, timing of restimulation, and cellular context [47,48,49]. Accordingly, the memory-like responses observed after LPS priming in this study resemble features previously described in studies of trained immunity in innate immune cells [25,48,50]. β-glucan is likewise a well-established inducer of memory-like responses through Dectin-1-mediated metabolic and epigenetic reprogramming [51,52]. The distinct responses observed between neonatal and adult neutrophils may reflect developmental differences in receptor expression, intracellular signaling efficiency, or metabolic adaptability [13,30,53]. Nitric oxide (NO) signaling may represent an additional contributor to these developmental differences, as NOS-derived NO can modulate neutrophil ROS production, antimicrobial functions, and maturation [54,55]. However, NO-dependent mechanisms were not assessed in the present study and require further investigation. Previous studies investigating neonatal innate immunity consistently reported reduced production of IL-12p70, type I interferons, and IFN-γ compared with adults [13,56,57]. In contrast, neonatal innate immune cells have been shown to produce higher levels of IL-6 and IL-1β during infancy, with TLR-mediated responses favoring Th17 cell development and supporting host defense against extracellular pathogens [38,58,59,60]. In addition, several studies demonstrated that the significantly higher serum adenosine concentrations observed in neonates compared with adults are associated with enhanced cytokine production, including IL-6 [10,61]. In line with these findings, Wynn et al. reported that priming with a TLR4 agonist enhanced peritoneal neutrophil recruitment and increased oxidative burst activity, independently of adaptive immune signaling [62]. Furthermore, BCG-induced trained immunity has been reported to modify myeloid transcriptional programs and stimulate the expansion of granulocyte–macrophage progenitor populations in neonatal mice [63]. Together, these findings indicate that neonatal innate immune cells are capable of generating strong inflammatory responses when exposed to appropriate stimuli. Such mechanisms may partly compensate for the still-developing adaptive immune system during early life and could represent an important component of age-dependent host defense.

A shift toward aerobic glycolysis is considered a characteristic feature of memory-like cues, as it provides the energy and biosynthetic resources required for rapid inflammatory responses [64,65,66]. Our data support the idea that memory-like neutrophil responses are associated with metabolic adaptation toward glycolysis. This was reflected by increased expression of the glycolytic enzymes HK-2 and PFK1 together with higher lactate release after stimulation. Similar metabolic shifts have been described previously in trained innate immune cells, where enhanced glycolysis contributes to inflammatory mediator production and cytokine signaling [64,67]. A notable finding was that neonatal neutrophils showed a more pronounced glycolytic profile than adult neutrophils. This difference was evident after priming but could also be detected, to some extent, under unstimulated baseline conditions. Earlier studies have likewise reported that neonatal immune cells rely more strongly on glycolytic metabolism, which may be related to adaptation to the fetal environment and the specific immune requirements encountered shortly after birth [64,68,69]. Such metabolic reprogramming may directly modulate the epigenetic landscape, as glycolytic flux and lactate generation can affect activating histone modifications, including H3K4me3 and H3K4me1, thereby promoting sustained pro-inflammatory gene expression [70,71]. Although our results are consistent with enhanced inflammatory responsiveness, they do not directly demonstrate chromatin remodeling, and the contribution of epigenetic mechanisms therefore remains speculative and warrants further investigation. Together, these findings provide a basis for future studies investigating the interplay between metabolic adaptation and epigenetic regulation in memory-like inflammatory responses during early life.

Mechanistically, the induction of memory-like responses in innate immune cells has been linked to activation of the AKT/mechanistic target of rapamycin (mTOR) pathway [48,64]. In addition, recent murine studies demonstrated that innate memory responses can also be regulated through MAPK-dependent signaling, leading to ATF7 phosphorylation and reduction of the repressive histone marks [24,65,72]. In our study activation of the phosphatidylinositol-3-kinase (PI3K)/AKT pathway was detectable; however, the memory-like phenotypes observed in circulating neutrophils, particularly in neonatal neutrophils stimulated with LPS or β-glucan, were accompanied by increased ERK1/2 and NF-κB activation. ERK1/2 signaling regulates neutrophil activation, cytokine production, and reactive oxygen species generation and has also been implicated in innate memory responses following microbial stimulation [24,48,73]. Although previous studies did not consistently demonstrate increased ERK1/2 activation in neonatal compared with adult neutrophils, findings by Yan et al. showing elevated MAPK expression in neonatal mononuclear cells, together with elevated endogenous galectin-3 levels in neonates, support the concept of a relatively primed inflammatory phenotype in neonatal innate immune cells [74,75]. The observed association between ERK1/2 activation and enhanced cytokine production paralleled the memory-like inflammatory responses in circulating neutrophils.

Several limitations should be considered when interpreting these findings. As this study was designed to investigate mechanistic aspects of memory-like responses in isolated neonatal neutrophils under controlled *in vitro* conditions, the results may not fully reflect the complexity of the neonatal immune environment in vivo. In addition, adult neutrophils were isolated from peripheral venous blood, whereas neonatal neutrophils were obtained from umbilical cord blood. Therefore, developmental age cannot be fully disentangled from potential effects of blood compartment and the distinct physiological environment associated with cord blood. Although the exclusive inclusion of term neonates delivered by cesarean section minimized variability related to labor and vaginal delivery, the contribution of compartment-specific and perinatal factors cannot be completely excluded when interpreting neonatal–adult differences. Moreover, although mothers with chronic inflammatory, infectious, or immunological disorders were excluded, unmeasured maternal and perinatal factors may still have contributed to inter-individual variability. The adult cohort included both female and male donors; however, the study was not designed or powered to investigate sex-dependent differences. Therefore, sex-stratified analyses were not performed, and potential effects of biological sex on neutrophil responses cannot be fully excluded. The relatively limited number of biological replicates in some experiments represents an additional limitation; however, this reflects the challenges associated with obtaining sufficient neonatal samples and highlights the need for validation in larger cohorts. Finally, the duration of the experiments was necessarily limited because circulating neutrophils survive only for a relatively short time ex vivo. As a result, long-term persistence of the observed memory-like responses could not be assessed in this model. In addition, sequential, outcome-dependent enrollment of matched donor pairs may have affected the reported nominal significance levels and should be considered when interpreting the statistical findings.

Overall, our results indicate that stimulation with LPS or β-glucan can induce memory–like inflammatory features in human neutrophils. These changes were associated with enhanced inflammatory activity, increased glycolytic metabolism, and activation of ERK1/2 and NF-κB signaling pathways. Compared with adult neutrophils, neonatal cells showed a more pronounced response pattern after training, pointing to a considerable degree of innate immune adaptability during early life. The findings further suggest that neonatal neutrophils may participate more actively in inflammatory and antimicrobial defense responses than has previously been appreciated.

## 4. Materials and Methods

### 4.1. Study Population and Sample Collection

Umbilical cord/placental blood was collected from term neonates (37–41 completed weeks of gestation) delivered by cesarean section to mothers aged 18–45 years after obtaining written and verbal informed consent. Participation required the absence of any intended alternative use of the cord blood, such as private banking. Mothers with chronic inflammatory or infectious diseases, known immunological or autoimmune disorders, or treatment with immunomodulatory agents were excluded. Detailed donor-level clinical information, including the presence or absence of labor, birthweight, and the clinical indication for cesarean section, was not collected under the approved ethics protocol. Peripheral venous blood from healthy adult volunteers serving as controls was obtained at the Department of Neonatology following informed consent. Participants eligible for inclusion were between 18 and 50 years old, had no history of chronic inflammatory disease, and were capable of giving informed consent. The adult cohort consisted of approximately 23 independent donors, including 19 females (82.6%) and 4 males (17.4%). Individuals were excluded in cases of pregnancy, suspected fetal abnormalities, acute or persistent infections, diagnosed immune disorders, or current treatment with immunosuppressive agents. Blood collection was carried out under sterile clinical conditions, and no more than 20 mL of blood was obtained from each participant.

Blood samples were collected in EDTA-coated tubes (S-Monovette, Sarstedt; containing 1.6 mg/mL ethylenediaminetetraacetic acid) and processed without delay after withdrawal. Polymorphonuclear neutrophils were isolated from adult peripheral blood as well as umbilical cord blood according to the protocols outlined below. Cell isolation and all subsequent experimental procedures were performed at the Laboratory of Neonatal Immunology, Department of Neonatology, University Children’s Hospital Heidelberg, Germany, where the samples were analyzed in vitro.

### 4.2. Compliance with Ethical Standards

Written informed consent for study participation and publication of data was obtained from all adult participants and from the legal guardians of neonates. The study protocol was approved by the Ethics Committee of the Medical Faculty Heidelberg (project ID: S-410/2024; approval date: 10 January 2025). All procedures were conducted in accordance with institutional and national ethical guidelines and complied with the principles of the Declaration of Helsinki and its subsequent amendments.

### 4.3. Human Neutrophil Isolation

Peripheral venous blood from healthy adult donors as well as umbilical cord and placental blood samples were processed directly after collection for neutrophil isolation following established procedures [76]. Whole blood (10–20 mL) was diluted 1:1 with Dulbecco’s phosphate-buffered saline (DPBS; Sigma-Aldrich, Taufkirchen, Germany, Cat. D8537) supplemented with 2 mM EDTA (Sigma-Aldrich, Cat. E5134). The diluted samples were carefully layered onto 15 mL Lymphocyte Separation Medium 1077 (PromoCell, Heidelberg, Germany, Cat. C-44010) in 50 mL tubes and centrifuged for 25 min at 400× *g* at room temperature without brake. After centrifugation, the plasma phase together with the mononuclear cell layer was removed. The lower fraction containing granulocytes and erythrocytes was transferred into fresh tubes and subjected to erythrocyte lysis using 1× red blood cell (RBC: 1.55 M NH_4_Cl, 120 mM NaHCO_3_, 1 mM EDTA). Samples were centrifuged at 500× *g* for 5 min, resuspended, and the lysis procedure was repeated until only minimal red blood cell contamination remained visible. The granulocyte fraction was washed once in sterile DPBS and centrifuged again. Purified neutrophils were finally resuspended in DPBS, and cell numbers were determined using a Neubauer chamber before further experiments. For stimulation assays, neutrophils from adult blood and neonatal cord blood were cultured in RPMI 1640 medium (Gibco, Darmstadt, Germany, Cat. 11875093) containing 10% heat-inactivated fetal bovine serum (PeloBiotech, Planegg, Germany, Cat. PB-FCS-EU-0500), 1% penicillin/streptomycin (Sigma-Aldrich, Cat. P4333), and 1% amphotericin B (Sigma-Aldrich, Cat. A2942). Flow cytometry confirmed high neutrophil purity (>91%) in all preparations within the expected range (Supplementary Figure S1), consistent with previously published isolation methods [76,77].

### 4.4. Neutrophil Stimulation Protocol

Freshly isolated adult and neonatal neutrophils were subjected to a sequential stimulation protocol adapted from previous reports investigating memory–like responses [24,25,78]. Cells were seeded at 3 × 10^5^ cells per well in complete RPMI medium and incubated at 37 °C in 5% CO_2_. During the initial stimulation phase, neutrophils were exposed for 3 h either to LPS (100 ng/mL; *Escherichia coli* O55:B5, InvivoGen, Toulouse, France, Cat. tlrl-pb5lps) or β-glucan (10 µg/mL; *Candida albicans* derived; provided by Prof. Dr. David L. Williams, East Tennessee State University, Johnson City, TN, USA) (Figure 1A). The β-glucan was isolated from *C. albicans* SC5314 and prepared according to the protocol described by Kruppa and colleagues [79,80]; it consisted of water-insoluble particulate β-glucan with an approximate particle size of ~3 µm. After priming, supernatants were collected for short-term cytokine analysis, and cells were centrifuged (450× *g*, 5 min), washed twice, and resuspended in fresh culture medium for the subsequent resting phase. Cells were then kept overnight for approximately 15 h before the second stimulation step. On the following day, neutrophils received a second challenge with LPS (100 ng/mL). Samples intended for RNA analyses were collected after 6 h, whereas supernatants and protein lysates for cytokine and signaling studies were harvested after 12 h. To discriminate baseline responses from effects related to prior priming, two separate control groups were included. Unstimulated (US) cells remained in medium alone throughout the experiment, while unprimed (UP) cells were exposed only to the second LPS stimulation without prior priming. This design allowed comparison between baseline responses and responses altered by previous stimulation.

### 4.5. Antibodies

Most primary antibodies used in this study were obtained from Cell Signaling Technology (Leiden, Netherlands). The following antibodies and dilutions were applied: phospho-p44/42 MAPK (ERK1/2) (mouse, 1:2000; Cat. 9106), total p44/42 MAPK (ERK1/2) (mouse, 1:1000; Cat. 9107), phospho-NF-κB p65 (rabbit, 1:1000; Cat. 3033), phospho-Akt (Ser473) (rabbit, 1:1000; Cat. 9271), total Akt (rabbit, 1:1000; Cat. 9272), and β-actin (mouse, 1:1000; Cat. 3700). β-actin served as loading control where appropriate. HRP-conjugated secondary antibodies against rabbit and mouse IgG were purchased from Dianova (Hamburg, Germany) and used at a dilution of 1:10,000.

### 4.6. Total Protein Quantification

Protein concentrations in cell lysates were measured using the Pierce™ 660 nm Protein Assay (Thermo Fisher Scientific; Darmstadt, Germany, Cat. 22662) together with the Ionic Detergent Compatibility Reagent (Cat. 22663). Samples, blanks and standards were loaded into 96-well plates using 10 µL per well. After addition of assay reagent and incubation according to the manufacturer’s instructions, absorbance was measured at 660 nm with a Bio-Rad (Feldkirchen, Germany) iMark plate reader. Protein concentrations were calculated using a standard curve generated under identical conditions.

### 4.7. SDS–PAGE and Immunoblotting

Cells were lysed on ice using RIPA buffer containing 50 mM Tris-HCl (pH 8.0), 150 mM NaCl, 1% NP-40, 0.5% sodium deoxycholate, and 0.1% SDS supplemented with protease and phosphatase inhibitors (Thermo Fisher Scientific; Cat. A32959). Following centrifugation at 12,000× *g* for 30 min at 4 °C, supernatants were collected and stored at −80 °C.

For immunoblot analysis, lysates were mixed with Laemmli buffer and heated for 5 min at 95 °C. Equal protein amounts were separated on 10% SDS-polyacrylamide gels and transferred onto PVDF membranes. Membranes were blocked in TBST containing 1% bovine serum albumin and incubated overnight with primary antibodies at 4 °C. After washing, HRP-linked secondary antibodies were applied for 1 h at room temperature. Detection was performed using enhanced chemiluminescence (WesternBright Sirius Kit; Biozym, Hessisch Oldendorf, Germany, Cat. 541020), and signals were recorded using a Bio-Rad ChemiDoc™ XRS+ system. Band intensities were analyzed with Image Lab software (Bio-Rad, version 6.0.1).

### 4.8. Flow Cytometry

Neutrophil purity was evaluated by flow cytometry. After exclusion of debris and doublets using FSC/SSC characteristics and FSC-H versus FSC-A gating, neutrophils were identified as CD66b^+^CD11b^+^ cells using FITC-conjugated anti-CD66b (clone G10F5; BioLegend, Amsterdam, The Netherlands) and PE-conjugated anti-CD11b (clone ICRF44; BioLegend). Purity consistently exceeded 91% in all samples analyzed. Measurements were performed on a FACSymphony A1 flow cytometer (BD Biosciences, Heidelberg, Germany), and data were processed with FlowJo software (version 10.10.0).

### 4.9. Enzyme-Linked Immunosorbent Assay (ELISA)

Cytokine and chemokine levels in culture supernatants were determined by ELISA kits. Prior to measurement, samples were centrifuged at 10,000× *g* for 5 min at 4 °C to remove residual debris and then stored at −80 °C until analysis. Commercial ELISA kits were used to quantify IL-6 (BioLegend; Cat. 430501), IL-8 (Cat. 431501), IL-1β (Cat. 437004), CXCL1/GROα (Bio-Techne; Wiesbaden, Germany, Cat. DY275), and MCP-1/CCL2 (Invitrogen; Darmstadt, Germany, Cat. 88-7399-88). All measurements were performed according to the manufacturers’ instructions and previously established experimental procedures [81,82].

Absorbance was recorded at 450 nm with reference correction at 570 nm using a Bio-Rad iMark microplate reader. Cytokine and chemokine concentrations were calculated from standard curves and expressed as pg/mL. Measurements were performed following both primary (3 h; priming) and secondary (12 h) stimulation time points.

### 4.10. Quantification of Reactive Oxygen Species (ROS)

Human adult and neonatal neutrophils were seeded at a density of 3 × 10^5^ cells per well in black, clear-bottom 96-well plates and stimulated according to the experimental setup described above (Figure 2A). ROS production was measured 6 h after the second stimulation with 100 ng/mL LPS using a fluorometric 2′,7′-dichlorodihydrofluorescein diacetate (DCFDA)-based assay (Cellular ROS Assay Kit; Abcam, Cambridge, UK, Cat. ab113851).

For detection, cells were incubated with 20 µM DCFDA for 30 min at 37 °C in the dark. Following incubation, excess dye was removed by washing with freshly prepared 1× assay buffer provided in the kit, and cells were resuspended in 1× supplemented assay buffer consisting of assay buffer supplemented with 10% FCS. Intracellular esterases convert DCFDA into a non-fluorescent intermediate, which is subsequently oxidized by reactive oxygen species to form the fluorescent product 2′,7′-dichlorofluorescein (DCF). Fluorescence was recorded using a Fluoroskan FL microplate reader (Thermo Fisher Scientific) at excitation and emission wavelengths of 485 nm and 535 nm, respectively. Results were expressed as relative fluorescence units (RFU), corresponding to relative intracellular ROS levels.

### 4.11. Quantification of Lactate Levels

Lactate concentrations in cell culture supernatants were determined using a colorimetric L-lactate assay kit (ScienCell Research Laboratories; San Diego, CA, USA, Cat. 8308) following the manufacturer’s instructions as described [67,82]. A working standard (4 mM) was prepared by diluting the provided L-lactate standard in assay buffer. A series of standards was generated by sequential two-fold dilutions in assay buffer to obtain a calibration curve, with assay buffer alone serving as the blank. Standards, samples, and blanks were dispensed in duplicate into a 96-well plate (50 µL per well). For enzymatic detection, 2 µL of the reconstituted enzyme mixture was added to each well, followed by 50 µL of substrate solution. The plate was incubated for 20 min at room temperature in the dark. Absorbance was measured at 490 nm using an iMark microplate reader, and lactate concentrations in the samples were calculated based on the standard curve.

### 4.12. cDNA Synthesis and Quantitative Real-Time PCR

Total RNA was extracted from human polymorphonuclear neutrophils 6 h after secondary stimulation with 100 ng/mL LPS using TRIzol™ Reagent (Invitrogen; Cat. 15596018), as described previously [47,82]. RNA quantity and purity were measured using a DS11 FX+ spectrophotometer (DeNovix, Wilmington, DE, USA). Complementary DNA (cDNA) was generated from the isolated RNA with the High-Capacity cDNA Reverse Transcription Kit (Applied Biosystems; Darmstadt, Germany, Cat. 4368814) following the manufacturer’s protocol. Quantitative real-time PCR (qPCR) analyses were carried out on a StepOnePlus Real-Time PCR System (Applied Biosystems) to determine expression levels of selected genes. The primer sequences used were as follows: PFK1 (forward: AGCGTTTCGATGATGCTTCAG; reverse: GGAGTCGTCCTTCTCGTTCC), HK-2 (forward: GAGTTTGACCTGGATGTGGTTGC; reverse: CCTCCATGTAGCAGGCATTGCT), NF-κB p65 (RELA) (forward: TGAACCGAAACTCTGGCAGCTG; reverse: CATCAGCTTGCGAAAAGGAGCC), ACTB (forward: CATCGAGCACGGCATCGTCA; reverse: TAGCACAGCCTGGATAGCAAC), and B2M (forward: ACTGAATTCACCCCCACTGA; reverse: CCTCCATGATGCTGCTTACA). Relative gene expression was normalized against the housekeeping genes ACTB and B2M, and fold changes were calculated using the 2^−^^ΔΔC^_T_ method [83].

### 4.13. Assessment of Cell Viability and Cytotoxicity

Cell viability in adult and neonatal human neutrophils was assessed 12 h after the secondary stimulation with 100 ng/mL LPS by using the trypan blue exclusion assay. Cells taking up trypan blue were considered non-viable, whereas viable cells excluded the dye. For analysis, 100 cells were counted per microscopic field, and the proportion of trypan blue–positive cells was determined. Four independent fields per condition were analyzed, and results were expressed accordingly (Supplementary Figure S4B). In addition, cell viability was assessed using the Resazurin Cell Viability Assay (Abcam; Cat. ab129732). Neutrophils (2 × 10^5^ cells/well) were seeded in 96-well plates, treated according to the experimental protocol, and incubated with the resazurin reagent for 4 h at 37 °C in a humidified atmosphere containing 5% CO_2_. Absorbance was measured at 570 nm using a Bio-Rad iMark microplate reader (Supplementary Figure S4D).

To assess cytotoxicity, cells were seeded at a density of 5000 cells per well and treated as described in the experimental protocol. Cytotoxicity was determined using a colorimetric Cell Cytotoxicity Assay Kit (Abcam; Cat. ab112118). Briefly, 20 µL of assay reagent was added to each well and incubated for 4 h at 37 °C in a humidified atmosphere containing 5% CO_2_, protected from light. Absorbance was measured at 570 nm using a Bio-Rad iMark microplate reader (Supplementary Figure S4C).

Data were expressed as relative viability or cytotoxicity and normalized to unprimed adult controls, which were set to 100%.

### 4.14. Statistical Analysis

Data visualization and statistical analyses were carried out using GraphPad Prism (v8.0.2; GraphPad Software) and SigmaPlot (v12.0; Systat Software GmbH). Unless otherwise indicated, n represents the number of independent biological donor pairs (one adult peripheral blood donor matched with one neonatal umbilical cord blood donor) and does not refer to technical replicates. Data normality was assessed using the Shapiro–Wilk test. Depending on the experimental design and data distribution, comparisons were performed using paired Student’s *t*-test or Wilcoxon matched-pairs signed-rank test (two-group comparisons), one-way ANOVA with Holm–Šidák multiple-comparisons post hoc test or Kruskal–Wallis test with Dunn’s post hoc test (multiple-group comparisons), and mixed-effects models (REML) with Šidák-adjusted multiple comparisons for factorial analyses. For small-sample datasets, non-parametric aligned rank transform (ART) analysis was used, followed by Mann–Whitney U and Wilcoxon signed-rank tests for pairwise comparisons. The specific statistical test used for each dataset is indicated in the corresponding figure legends. Data are presented as scatter dot plots with mean ± standard error of the mean (SEM). A *p*-value of less than 0.05 was considered statistically significant.

## 5. Conclusions

These findings support the concept that neutrophils, despite their short lifespan and terminal differentiation status, are capable of functional adaptation beyond immediate innate effector responses. We show that neonatal neutrophils exhibit pronounced memory-like inflammatory responses following LPS or β-glucan exposure, characterized by enhanced inflammatory mediator production and increased glycolytic activity, and accompanied by increased ERK1/2 and NF-κB activation. The stronger responses observed in neonatal compared with adult neutrophils further indicate that innate immune function during early life possesses substantial functional plasticity. Given the importance of innate immunity during the neonatal period, further studies are required to determine the biological relevance and potential clinical implications of memory-like responses of neutrophils in early life.

## Data Availability

The datasets used in the current study are available from the corresponding author on reasonable request.

## Supplementary Materials

The following supporting information can be downloaded at: https://www.mdpi.com/article/10.3390/ijms27167268/s1.

## Abbreviations

The following abbreviations are used in this manuscript:

AKT	Protein kinase B
CCL2	C-C motif chemokine ligand 2
CXCL1	Chemokine (C-X-C motif) ligand 1
ERK 1/2	Extracellular signal–regulated kinase 1/2
IFN	Interferon
IL	Interleukin
HK-2	Hexokinase-2
LPS	Lipopolysaccharide
LTA	Lipoteichoic acid
MAPK	Mitogen-activated protein kinase
NET	Neutrophil extracellular traps
NF-κB	Nuclear factor kappa B
NO	Nitric oxide
oxLDL	Oxidized low-density lipoprotein
PAMPs	Pathogen-associated molecular patterns
PFK1	Phosphofructokinase-1
PI3K	Phosphatidylinositol-3-kinase
PRRs	Pathogen recognition receptors
ROS	Reactive oxygen species
TLR	Toll-like receptor
UP	Unprimed
US	Unstimulated
