# Supplementary material for: LPS and β-Glucan Induce Differential Memory-like Inflammatory Responses with Enhanced Reactivity in Neonatal Neutrophils Compared to Adults
Source: Int J Mol Sci. 2026 Aug 14;27(16):7268. doi: 10.3390/ijms27167268 (PMC13513832; doi:10.3390/ijms27167268)
Supplement: Supplementary file 1 [file ijms-27-07268-s001.zip › ijms-4474662-supplementary.pdf]

Supplementary Figures

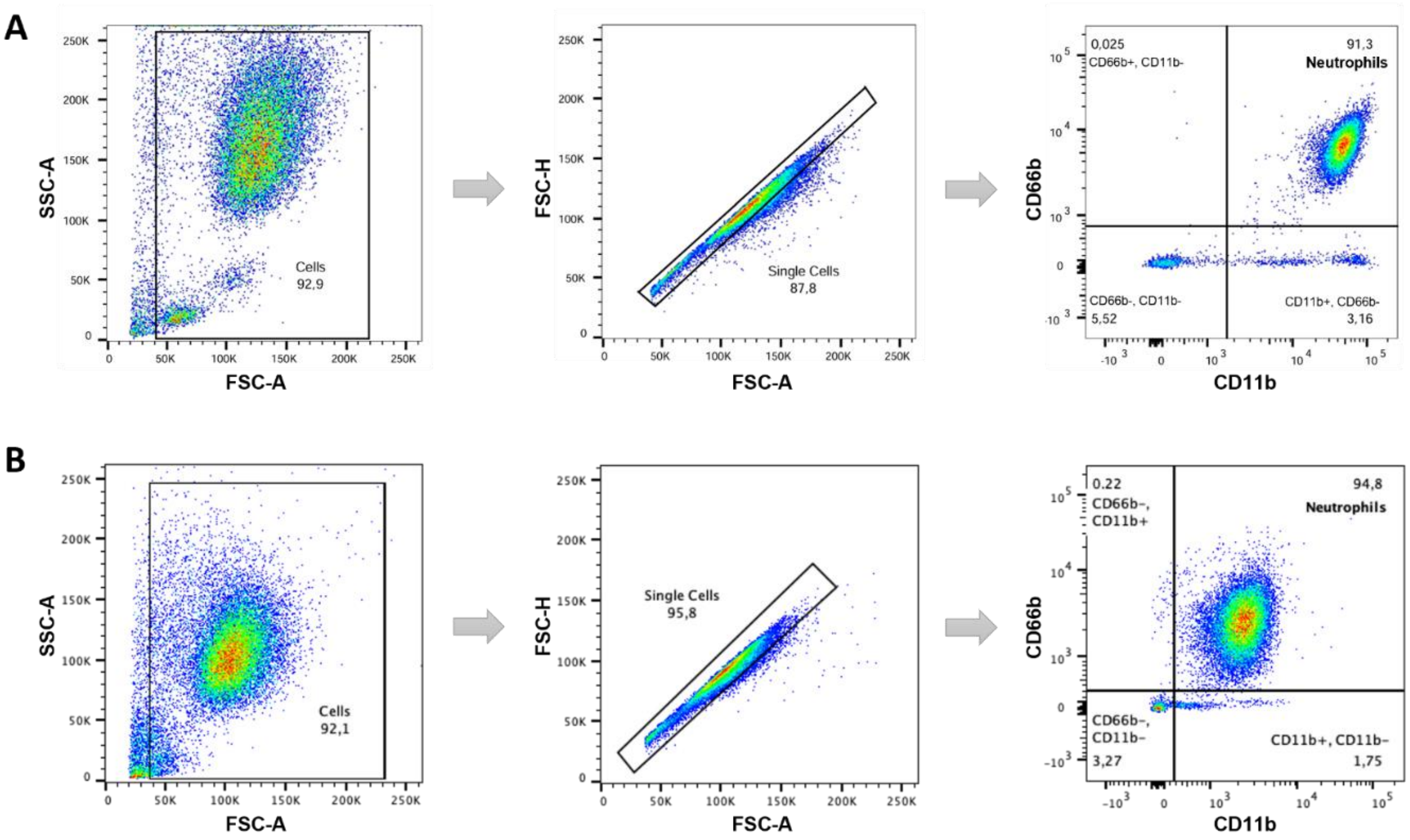

Supplementary Figure S1. Purity assessment and representative flow cytometry gating strategy for adult and neonatal neutrophils. Purified neutrophil populations isolated from adult peripheral blood and umbilical cord/placental blood were evaluated for purity by flow cytometry. Representative dot plots illustrate the gating strategy used to identify polymorphonuclear neutrophils. (A) Adult and (B) neonatal neutrophils were defined based on surface expression of CD66b (FITC-conjugated anti-human CD66b, clone G10F5; BioLegend) and CD11b (PE-conjugated anti-human CD11b, clone ICRF44; BioLegend), with $CD66b^{+}CD11b^{+}$ cells defined as the neutrophil population.

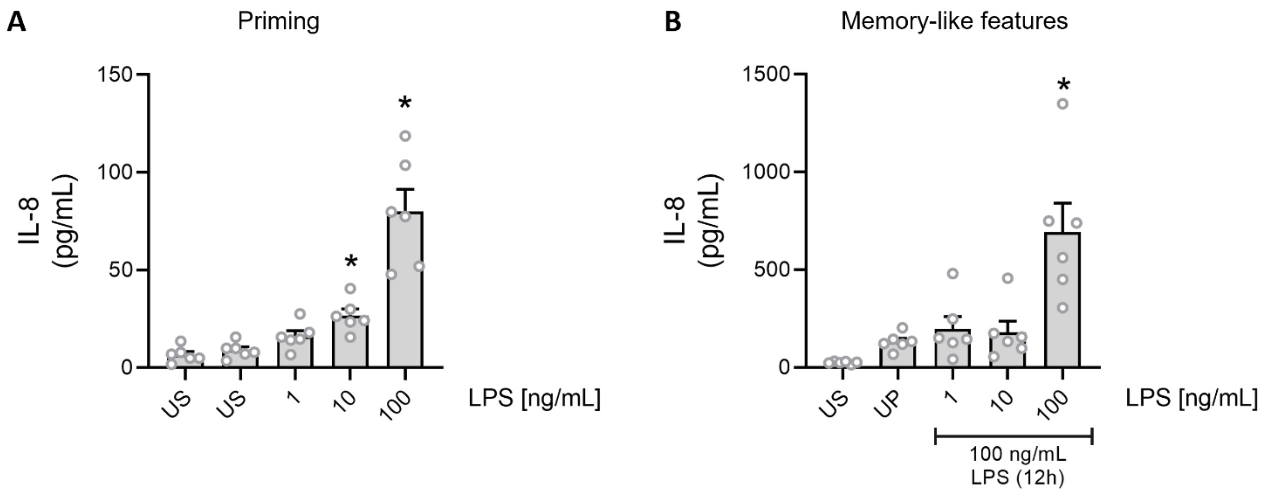

Supplementary Figure S2. Dose-dependent effects of LPS priming on the induction of memory-like inflammatory responses in primary human adult neutrophils. Cells were initially primed with LPS (1, 10, or 100 ng/mL) for 3 h to assess the immediate inflammatory response (A, n = 6) or, following a 15 h resting period, were re-challenged with LPS (100 ng/mL) for an additional 12 h to evaluate memory-like (B, n = 6) inflammatory responses. IL-8 concentrations in cell culture supernatants were quantified by ELISA after the initial priming phase and after secondary LPS stimulation. Data were presented as scatter dot plots, mean ± SEM, Kruskal-Wallis (Dunn's multiple-comparison post-hoc test), *p<0.05, * indicating statistical significance compared to unstimulated (US) control (A); *p<0.05, * indicating statistical significance compared to unprimed (UP) control (B).

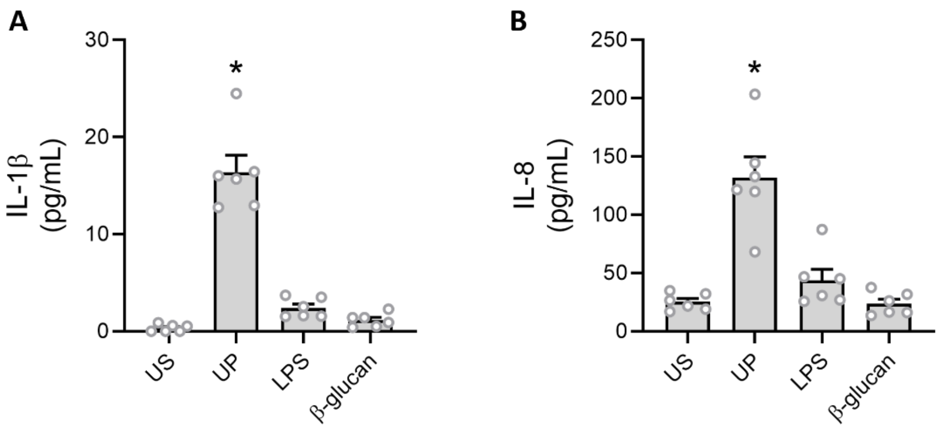

Supplementary Figure S3. Cytokine production following the resting phase in primed human adult neutrophils. Cells were initially primed with LPS (100 ng/mL) or β-glucan (10 μg/mL) for 3 h. Following priming, culture medium was replaced, and cells were maintained under resting conditions for a total period corresponding to the original experimental protocol (15 h resting plus the subsequent 12 h secondary challenge interval), without re-stimulation. In parallel, an unprimed control group was challenged with LPS (100 ng/mL) for 12 h to serve as a reference for the primary inflammatory response. Concentrations of IL-1β (A, n = 6) and IL-8 (B, n = 6) in culture supernatants were determined by ELISA. Data were presented as scatter dot plots, mean ± SEM, Kruskal-Wallis (Dunn's multiple-comparison post-hoc test), *$p<0.05$, * indicating statistical significance compared to unstimulated (US) control.

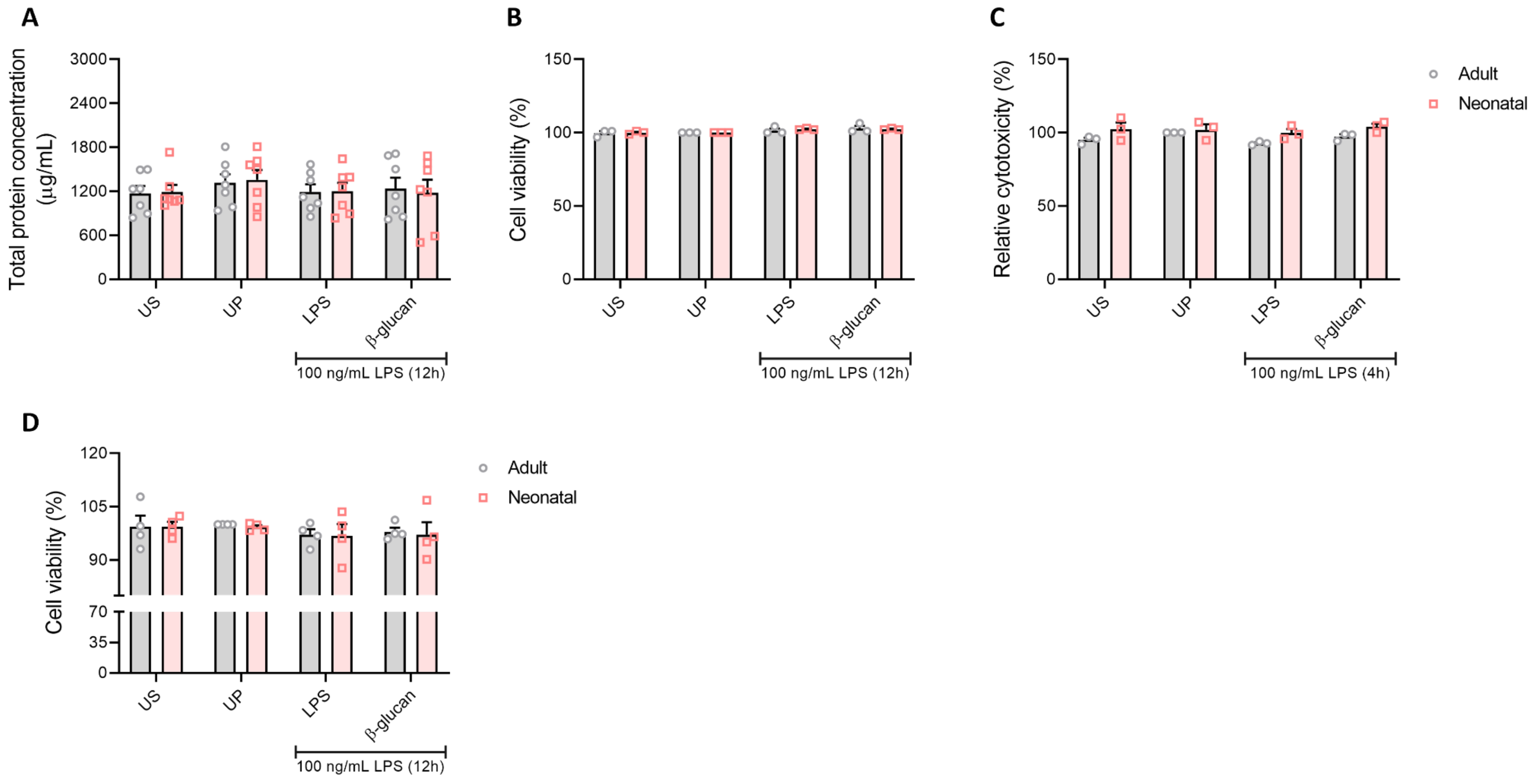

Supplementary Figure S4. Protein content, viability, and cytotoxicity of adult and neonatal neutrophils after priming and secondary stimulation. Cells were initially primed with LPS (100 ng/mL) or β-glucan (10 µg/mL) for 3 h, followed by a resting period of approximately 15 h and subsequent restimulation with LPS (100 ng/mL) for 12 h. (A) Total protein concentration (n = 7) was measured using the Pierce™ 660 nm Protein Assay and analyzed using a mixed-effects model (REML), with group, condition, and their interaction included as fixed effects and biological sample included as the random effect. Neutrophil viability assessed after LPS restimulation using trypan blue exclusion (B, n = 3) and resazurin assay (D, n = 4), whereas (C) cell cytotoxicity (n = 3) determined using a commercially available colorimetric Cell Cytotoxicity Assay Kit (unprimed adult cells assigned as 100%). Data were

presented as scatter dot plots, mean ± SEM. Panels B–D were analyzed using an aligned rank transform (ART) non-parametric analysis to assess the effects of developmental group, experimental condition, and their interaction.

Supplementary Tables

Parameter	Adult	Neonatal	Statistical test	Adjusted p-value
**IL-6** *(pg/mL)*	3,664 ± 1,072	4,422 ± 0,992	Paired t-tests	p = 0,646
**IL-8** *(pg/mL)*	32,076 ± 2,650	40,206 ± 7,699	Paired t-tests	p = 0,330
**IL-1β** *(pg/mL)*	0,405 ± 0,262	0,208 ± 0,208	Wilcoxon signed-rank test	p = 1,000
**CCL2** *(pg/mL)*	16,097 ± 1,842	28,178 ± 2,570	Paired t-tests	**p < 0,001**
**CXCL1** *(pg/mL)*	7,043 ± 0,645	5,364 ± 0,860	Paired t-tests	p = 0,123
**ROS** *(RFU)*	0,655 ± 0,028	0,975 ± 0,025	Paired t-tests	**p < 0,001**
**Lactate** *(mM)*	1,282 ± 0,075	2,588 ± 0,100	Paired t-tests	**p < 0,001**

Supplementary Table S1. Baseline unstimulated (US) adult and neonatal values were compared for each individual readout using paired statistical tests because samples were obtained from matched donor pairs. Paired t-tests were applied for normally distributed data, whereas the Wilcoxon matched-pairs signed-rank test was used when the normality assumption was not met. Data are presented as mean ± SEM.
